# Repeated *Schistosoma japonicum* Infection Following Treatment in Two Cohorts: Evidence for Host Susceptibility to Helminthiasis?

**DOI:** 10.1371/journal.pntd.0002098

**Published:** 2013-03-07

**Authors:** Elizabeth J. Carlton, Alan Hubbard, Shuo Wang, Robert C. Spear

**Affiliations:** 1 Environmental Health Sciences, School of Public Health, University of California, Berkeley, California, United States of America; 2 Biostatistics, School of Public Health, University of California, Berkeley, California, United States of America; Imperial College London, United Kingdom

## Abstract

**Background:**

In light of multinational efforts to reduce helminthiasis, we evaluated whether there exist high-risk subpopulations for helminth infection. Such individuals are not only at risk of morbidity, but may be important parasite reservoirs and appropriate targets for disease control interventions.

**Methods/Principal Findings:**

We followed two longitudinal cohorts in Sichuan, China to determine whether there exist persistent human reservoirs for the water-borne helminth, *Schistosoma japonicum*, in areas where treatment is ongoing. Participants were tested for *S. japonicum* infection at enrollment and two follow-up points. All infections were promptly treated with praziquantel. We estimated the ratio of the observed to expected proportion of the population with two consecutive infections at follow-up. The expected proportion was estimated using a prevalence-based model and, as highly exposed individuals may be most likely to be repeatedly infected, a second model that accounted for exposure using a data adaptive, machine learning algorithm. Using the prevalence-based model, there were 1.5 and 5.8 times more individuals with two consecutive infections than expected in cohorts 1 and 2, respectively (p<0.001 in both cohorts). When we accounted for exposure, the ratio was 1.3 (p = 0.013) and 2.1 (p<0.001) in cohorts 1 and 2, respectively.

**Conclusions/Significance:**

We found clustering of infections within a limited number of hosts that was not fully explained by host exposure. This suggests some hosts may be particularly susceptible to *S. japonicum* infection, or that uncured infections persist despite treatment. We propose an explanatory model that suggests that as cercarial exposure declines, so too does the size of the vulnerable subpopulation. In low-prevalence settings, interventions targeting individuals with a history of *S. japonicum* infection may efficiently advance disease control efforts.

## Author Summary

Approximately 1 billion people are infected with one or more helminthes – a class of parasites that can impair physical, mental and economic development. We are interested in whether there exist groups who are repeatedly infected with helminthes over time in areas where treatment is ongoing. Such individuals may be at risk of morbidity and may also serve as parasite reservoirs, making them appropriate targets for disease control programs. We followed two cohorts in rural Sichuan, China in order to evaluate whether the same individuals were repeatedly infected with the water-borne helminth, *Schistosoma japonicum*. Each participant was tested for infection at enrollment and two follow-up points – all infections were promptly treated. We conducted detailed interviews to assess exposures to *S. japonicum*. We found infections repeatedly occurred in a subgroup of individuals and this clustering of infections was only partly explained by differences in exposure. This suggests some individuals may be particularly susceptible to *S. japonicum* infection. Further exploration of the interplay of exposure and susceptibility suggest that as exposure declines, so too does the fraction of the population vulnerable to infection. Helminth control programs that target people with a history of infection may efficiently reduce helminth infections and morbidity.

## Introduction

Recent multinational efforts to control and eliminate helminthiasis have the potential to dramatically reduce morbidity among the rural poor [1], [2]. Approximately one billion people are infected with one or more helminthes and the health impacts of these infections, including impaired growth, cognitive development and work capacity are substantial and poverty reinforcing [3]–[5]. Population-level interventions are the recommended strategy in areas where infection prevalence and morbidity are high [6], but as infections decline, how should limited disease control resources be allocated in order to sustain disease control achievements?

We are interested in whether there exist high-risk subpopulations for helminth infection, as such individuals may not only be particularly vulnerable to morbidity, they may also play a key role in sustaining transmission in regions where control efforts have reduced but not eliminated helminthiasis [7]. In many infectious disease transmission systems, a few individuals are responsible for a disproportionate number of future infections: control efforts targeting such superspreaders can efficiently reduce disease transmission compared to randomly allocated or population-based control efforts [8], [9]. In the case of helminthiasis, helminthes typically are aggregated in a population such that at any point in time, a few individuals harbor a large number of worms and therefore may be responsible for a large number of future infections [9]. If the same individuals are repeatedly infected, this suggests the presence of high-risk groups for helminthiasis – groups that may serve as persistent parasite reservoirs in the presence of on-going treatment and control efforts. Prior research suggests such high-risks groups may exist: for example, past infection with the water-borne helminth, *Schistosoma sp.* is a positive predictor of subsequent infection [10]–[13].

What mechanisms might promote the aggregation of infections in a few individuals? The cross-sectional clustering of helminthes in a population has largely been attributed to differential pathogen exposure – highly exposed individuals are most likely to harbor greater pathogen loads [9], [14]. If we assume an individual's exposure is relatively constant over time, we expect the same, highly exposed individuals will be repeatedly infected over time. Host susceptibility to infection may also favor repeated infections in a particular subpopulation. Host genetics play a role in susceptibility to soil-transmitted helminthiases and schistosomiasis, likely via variations in genes regulating immune function, including, in the case of *Schistosoma sp.*, Th2 response [15]–[18]. In contrast to exposure and host-susceptibility, exposure-dependent immunity should protect highly infected individuals at a given time point from subsequent infection, resulting in a disaggregation of infections across the population over time. Age-dependent immunity should concentrate infections in vulnerable age groups, leading to time-limited membership in high-infection subpopulations.

We examined longitudinal patterns of infection with the water-borne helminth, *S. japonicum*, in two cohorts in order to assess the aggregation of infections in the same individuals over time and, if present, the extent to which aggregation can be attributed to exposure vs. host-susceptibility.

## Methods

We followed two cohorts of rural residents in Sichuan, China drawn from hilly regions where schistosomiasis is associated with irrigated agriculture. Cohort 1 is composed of 424 individuals from 10 villages located in Xichang County, in southwest Sichuan, monitored from 2000 to 2006, a region where *S. japonicum* infection prevalence and intensity has historically been high. Cohort 2 is composed of 400 individuals from 27 villages in 2 counties in Sichuan province where schistosomiasis reemerged following reduction of human infection prevalence below 1%, a benchmark for schistosomiasis transmission control [19]. Individuals in the second cohort were monitored from 2007 to 2010. In each cohort, we tested all participants for *S. japonicum* infection at enrollment, treated all infections and conducted detailed exposure assessments. Participants were tested for incident infection at two follow-up points.

### Cohort 1

In fall 2000, we conducted *S. japonicum* exposure and infection surveys in 20 villages in Xichang County [20]. All residents were invited to participate in *S. japonicum* infection surveys (individuals age 4–60 were targeted, but infection testing was open to people of any age). A 25% random sample of residents, stratified by village and occupation, was interviewed about water contact behaviors at the same time as the infection surveys. Individuals were asked to report the frequency and duration of contact with surface water sources while conducting the following activities: washing clothes or vegetables, washing agricultural tools, washing hands and feet, playing or swimming, irrigation ditch operation or maintenance, rice planting, rice harvesting, and fishing; for each month from April to October (Supporting Information S1 in Text S1). Infection surveys were repeated in 2002 and 2006 in ten villages with high infection prevalence in 2000 (range 12.9 to 72.3%). This cohort includes all individuals from the 10 follow-up villages who completed the water contact interview and were tested for infection all three years. Infection status and intensity at enrollment did not differ between cohort members that were lost to follow-up and those with complete data, but those who were lost to follow-up reported less water contact and were younger, on average. Details of cohort selection and retention are provided in Figure S1 and Table S1 in Text S1.

### Cohort 2

In fall 2007, a cross sectional survey was conducted in 53 villages in three counties where *S. japonicum* reemerged following attainment of national transmission control criteria [21]. All residents age 6 to 65 were invited to participate in *S. japonicum* infection surveys. In May 2008, a magnitude 7.9 earthquake in Sichuan severely impacted one of the three selected counties, forcing us to limit follow-up studies to the two other counties. For efficiency, water contact behaviors were assessed using a stratified random sample of individuals based on 2007 infection status. All individuals who tested positive for *S. japonicum* in 2007, and, for each infected person, five people randomly drawn from the same village who tested negative for *S. japonicum* in 2007, were selected for participation in a survey of water contact behaviors. Interviews about water contact patterns were conducted monthly, from June to October 2008. At each interview, participants were asked to report the frequency and duration of water contact activities in the past two weeks including washing laundry, washing vegetables, washing agricultural tools, washing hands or feet, playing or swimming, ditch cleaning and repair, rice planting, rice harvesting, fishing, and collecting water for drinking or cooking. During the first interview, participants were also asked to report water contact behaviors during the May rice planting season, as, due to earthquake relief efforts, no interviews were conducted in May. Nobody reported water contact while collecting water for drinking and cooking, and this behavior was excluded from analyses. For comparability with cohort 1, washing laundry and washing vegetables were combined into a single water contact measure. Participants were tested for *S. japonicum* infection again in 2008 and 2010.This cohort includes all individuals who were tested for infection all three years and completed the water contact interview. As was the case for cohort 1, baseline infection status and intensity did not differ between cohort 2 members that were lost to follow-up and those with complete data, but those who were lost to follow-up reported less water contact, were more likely to be male and were younger, on average. Details of cohort selection and retention are provided in Figure S2 and Table S1 in Text S1.

As some members of cohort 2 did not complete all monthly water contact interviews, missing water contact measures were imputed using multiple imputation by chained equations [22], [23]. Multiple imputation avoids bias presented by the exclusion of incomplete cases. Imputation is based on the assumption that data are missing at random, and that missing data can be explained by other measured variables [24]. We imputed water contact minutes by month and activity using all other water contact measures, as well as age, sex, and village of residence. During the monthly interviews, participants were also asked to report the number of days they spent outside of their village and distance traveled in the past month. As travel may influence water contact patterns, travel was also included in the set of existing data used to impute missing values. The duration of water contact was imputed using predictive mean matching. Because nobody reported water contact from fishing in October or rice harvesting in June, all individuals missing these variables were assumed to have zero water contact for this exposure. Participants with one or more missing values were more likely to be younger and live in county 1, but did not otherwise differ substantially from participants with complete data (Table S2 in Text S1).Ten imputed datasets were generated. We calculated the mean of each imputed value for use in the predictive models described below. Before imputation, 5.0% of the water contact measures were missing: 71 participants (18%) did not complete all monthly interviews and 13 participants (3%) were interviewed each month but did not answer all survey questions.

All questionnaires in both cohorts were administered in the local dialect by trained staff at the Institute of Parasitic Diseases (IPD), Sichuan Center for Disease Control and Prevention and the county Anti-schistosomiasis Control stations.

### *S. japonicum* infection measures

During each infection survey, participants were asked to submit three stool samples, one each from three consecutive days. Each sample was analyzed using the miracidia hatching test: approximately 30 grams of stool was filtered, suspended in aqueous solution and examined for miracidia according to Chinese Ministry of Health protocols [25]. In addition, one sample from each participant was analyzed using the Kato-Katz thick smear procedure: three slides were prepared using 41.7 mg homogenized stool per slide and examined for *S. japonicum* eggs by trained technicians [26]. Infection intensity, in eggs per gram of stool (EPG), was calculated as the total number of *S. japonicum* eggs divided by the total sample weight. In 2002, only one stool sample was collected per person in cohort 1, and this sample was analyzed using both the miracidia hatching test and the Kato-Katz thick smear procedure. After each infection survey, all individuals testing positive for *S. japonicum* were promptly notified and provided treatment with 40 mg/kg praziquantel by health workers at the county anti-schistosomiasis control stations.

### Ethics

The research protocols and informed consent procedures and were approved by the Sichuan Institutional Review Board and the University of California, Berkeley, Committee for the Protection of Human Subjects. In cohort 1, all participants provided oral informed consent, documented by IPD staff, before participating in this study. Oral consent was obtained due to the high prevalence of illiteracy, and because the survey procedures used were similar to those used by IPD for schistosomiasis surveillance. In cohort 2, all participants provided written, informed consent before participating in this study. Minors provided assent and their parents or guardians provided written, informed permission for them to participate in this study.

### Statistical analysis

We examined the extent to which *S. japonicum* infections repeatedly occur in the same individuals in regions where schistosomiasis case detection and treatment is ongoing. For each cohort we defined three time points: baseline (*T_0_*), the first follow-up infection survey (*T_1_*) and the second follow-up infection survey (*T_2_*). We estimated the ratio of the observed proportion of the population with of two consecutive infections at *T_1_* and *T_2_* (*O_DI_*), to the predicted proportion of the population with two consecutive infections at *T_1_* and *T_2_* (*P_DI_*).

The simplest model of *P_DI_* is based solely on the probability of infection at *T_1_* and *T_2_*, such that 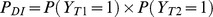 where 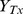 indicates *S. japonicum* infection status at time point *x*. Because all infections were treated at each time point, the probability of infection at *T_x_* is the incidence of infection from *T_(x-1)_* to *T_x_* multiplied by the elapsed time between *T_(x-1)_* and *T_x_*, which is equal to the prevalence of infection at *T_x_*. Note that at *T_0_*, we know the prevalence, but not the time elapsed since last treatment, which may vary by individual, and therefore can only estimate the probability of infection at *T_1_* and *T_2_*. Our estimates of infection probability assume all infections, defined as the presence of adult *S. japonicum* worm pairs, are detected and successfully treated at each time point. Using this prediction model, if 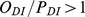, this suggests that there exists a subset of individuals that are repeatedly infected with *S. japonicum*.

A more complex model of *P_DI_* accounts for exposure, as individuals who are repeatedly infected may be those who are most highly exposed to *S. japonicum* cercariae. In this case, we estimate 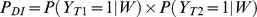 where 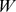 is *S. japonicum* cercarial exposure. Using this exposure-based prediction model, if 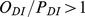, this suggests that *S. japonicum* infections repeatedly occur in a subset of individuals in the population for reasons not attributable to the exposure variables in the statistical model. *S. japonicum* cercarial exposure is determined by human behaviors that put people in contact with potentially contaminated water sources (primarily irrigation ditches and ponds), and by cercarial concentrations at the site of contact. We accounted for human behavior using questionnaire derived estimates of month- and activity-specific water contact duration. Cercarial concentration can vary over space and time due to the non-uniform distribution of the intermediate snail host and because cercarial shedding is affected by temperature, diurnal patterns and reservoir host species [27]–[29]. Currently, practical, field deployable methods for measuring cercarial concentrations are lacking. A mouse bioassay exists, in which sentinel mice are dermally exposed to surface water, then sacrificed and examined for *S. japonicum* worms, approximately 45 days post-exposure (allowing time for the parasite to mature inside the host). The mouse bioassay is not only resource intensive but, in low-prevalence settings, has limited sensitivity and, while new molecular methods offer promise, they have yet to be widely deployed [30], [31]. We used several proxies for cercarial concentration in our infection prediction models. We included village infection prevalence at T_0_, based on the assumption that villages with more infected individuals at enrollment have the potential for greater cercarial concentrations. In cohort 2, we also included county in the infection prediction model, as control measures which may impact cercarial concentration such as application of moluscicides are administered at the county level (all participants in cohort 1 are from a single county). To account for temporal variation in cercarial concentration, we included the year of infection testing. Additionally, we included age and sex to account for potential differences in the location of water contact (concentration) and the reporting of water contact activities (behavior) by age and sex.

The first step in estimating *P_DI_* requires a model that predicts infection status at a given time point based on exposure: 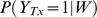. However, given the large number of predictor variables and the potentially complex, nonlinear relationships between exposure and infection, any single arbitrary parametric model one might choose will lead to an unknown degree of bias in the estimate of 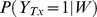 and, ultimately, *P_DI_* [32]. To minimize this problem we used a machine-learning algorithm, known as the Super learner as implemented in R [33]. In essence, this procedure estimates 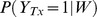 based on a convex combination of a number of different modeling algorithms (some simple parametric models, some highly data adaptive, generically called *learners*). In this case, the learners include random forests [34], k-nearest neighbor classification [35], elastic net regression [35], generalized linear models, stepwise regression and generalized boosted regression [36]. Cross-validation is used to determine the optimal combination of learners, that is the combination that maximizes the cross-validated fit. It has been shown that the Super learner estimate is asymptotically equivalent to the estimator that would come closest to the truth if the truth were known (called the Oracle selector), even if a very large number of competing models were used. In addition, in the unlikely case that the true model is a simple parametric model, then Super learner achieves nearly the same performance as a simple parametric estimation procedure (a parametric Oracle). From a practical point of view, Super learner replaces the usual ad hoc exploration of the adequacy and fit of various candidate models with a machine-based procedure that produces a robust, replicable, and theoretically defensible estimate.

We excluded from the set of exposure variables water contact variables for which <20% of the population reported any water contact. Models were fit separately for each cohort. An individual's infection probability was calculated for each year (*T_1_* and *T_2_*) using the selected model, and the probability of two consecutive infections was calculated as the product of the infection probabilities at *T_1_* and *T_2_*.

All estimates of observed and predicted infections were weighted to account for the stratified sampling used to assess water contact behavior. Each individual in the cohort was assigned a weight equal to the inverse probability of being sampled. Inference was estimated by calculating the probability of the observed number of consecutively infected individuals in the reweighted population (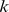). We assumed 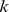 follows a binomial distribution 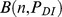 where 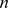 is equal to the number of individuals in the reweighted population and 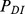 is the probability of two consecutive *S. japonicum* infections in an individual.

Statistical analyses were conducted using Stata12.0 and R 2.14.1 software.

## Results

The demographic characteristics of the two cohorts, reported water contact behaviors and the distribution of *S. japonicum* infections at enrollment are presented in Table 1. In the 10 villages from which cohort 1 was drawn, mean *S. japonicum* infection prevalence among all 1,801 residents surveyed was 46.9% (12.9 to 72.3% by village) and intensity, 46.0 EPG (1.1 to 107.9 EPG by village) at enrollment (*T_0_*). In the 27 villages from which cohort 2 was drawn, mean infection prevalence among all 1,608 individuals surveyed was 10.6% (1.5 to 42.9% by village) and intensity 2.6 EPG (0 to 10.6 EPG by village). Note that in 3 villages, infections were detected by the miracidia hatching test only, no eggs were detected by the Kato-Katz method, resulting in mean village infection intensities of 0 EPG. In both cohorts, adults were generally farmers with limited formal schooling. The percent of people reporting water contact, and the average duration of water contact varied by month, activity and cohort.

**Table 1:** Description of the two cohorts at enrollment (*T_0_*).

	Cohort 1*	Cohort 2†
Year of enrollment (*T_0_*)	2000	2007
*S. japonicum* infection prevalence in cohort villages at enrollment‡	46.9	10.6
Mean infection intensity in EPG in cohort villages at enrollment (SE)‡	46.0 (4.7)	2.6 (0.6)
% Female	51.9	57.3
Mean age at enrollment (SE)	31.3 (0.7)	45.6 (0.7)
% of adults reporting farming as their occupation	92.9	98.7
% of adults that have at least a middle school education	39.3	22.0
Mean water contact hours by month (% reporting any water contact)**		
April	28.4 (79.7)	–
May	5.4 (80.9)	44.8 (82.2)
June	7.7 (82.3)	11.4 (72.5)
July	4.8 (83.5)	4.6 (59.0)
August	4.9 (83.0)	3.8 (53.0)
September	5.4 (78.5)	18.6 (57.7)
October	2.9 (69.3)	2.1 (40.5)
Mean water contact hours by activity (% reporting any water contact)		
Washing vegetables or laundry	5.7 (23.6)	11.6 (60.3)
Irrigation ditch operation or maintenance	1.9 (54.7)	7.7 (40.5)
Fishing	0.4 (3.1)	0.4 (4.0)
Washing hands or feet	11.6 (63.0)	3.7 (87.0)
Harvesting rice	1.6 (4.2)	16.6 (43.0)
Planting rice	32.2 (70.3)	42.3 (66.5)
Swimming or playing	5.0 (16.5)	0.2 (9.5)
Washing agricultural tools	1.1 (29.0)	2.8 (72.3)

* Cohort 1 is composed of 424 residents from 10 villages in Xichang County, Sichuan, China where schistosomiasis was endemic, monitored from 2000 to 2006.

† Cohort 2 is composed of 400 residents from 27 villages in two counties in Sichuan, China where schistosomiasis reemerged following reduction of *S. japonicum* infection prevalence below 1%, monitored from 2007 to 2010.

‡ Prevalence and infection intensity estimates include all participants in village-wide infection surveys conducted at cohort enrollment: 1,801 individuals in 10 villages in cohort 1, 1,608 individuals in 27 villages in cohort 2.

** Participants were asked about water contact behaviors from the start of the rice planting season. In Xichang County (from which cohort 1 participants were drawn) rice planting begins in April, whereas in the two reemerging counties (from which cohort 2 participants were drawn) rice planting begins in May.

Infection prevalence and intensity at follow-up was low in both cohorts (Table 2). Notably, many individuals who tested positive for *S. japonicum* had no detectable eggs through the Kato-Katz examination: these individuals were positive via the miracidia hatching test only. In cohort 1, 30% and 27% of the individuals that tested positive for *S. japonicum* infection at *T_1_* and *T_2_*, respectively, had no detectable *S. japonicum* eggs on Kato-Katz examination. In cohort 2, 55% and 65% of infected individuals at *T_1_* and *T_2_*, respectively, had no detectable *S. japonicum* eggs on Kato-Katz examination.

**Table 2:** *S. japonicum* infection prevalence and intensity at follow-up.

	Cohort 1*		Cohort 2†	
	*T_1_*	*T_2_*	*T_1_*	*T_2_*
*S. japoincum* infection prevalence‡	33.1	10.3	7.7	7.8
Mean *S. japonicum* infection intensity‡	8.7	3.1	4.3	1.3

* Cohort 1 is composed of people from 10 villages where schistosomiasis is endemic. Participants were tested for *S. japonicum* infection in 2000 (*T_0_*), 2002 (*T_1_*) and 2006 (*T_2_*).

† Cohort 2 is composed of people from 27 villages in two counties where schistosomiasis reemerged following reduction of *S. japonicum* infection prevalence below 1%. Participants were tested for *S. japonicum* infection in 2007 (*T_0_*), 2008 (*T_1_*) and 2010 (*T_2_*).

‡ Infection prevalence and intensity were estimated for the source population, accounting for the stratified sampling used in enrolling cohort participants. Each individual in the cohort was assigned a weight equal to the inverse probability of being sampled.

**Figure 1:**
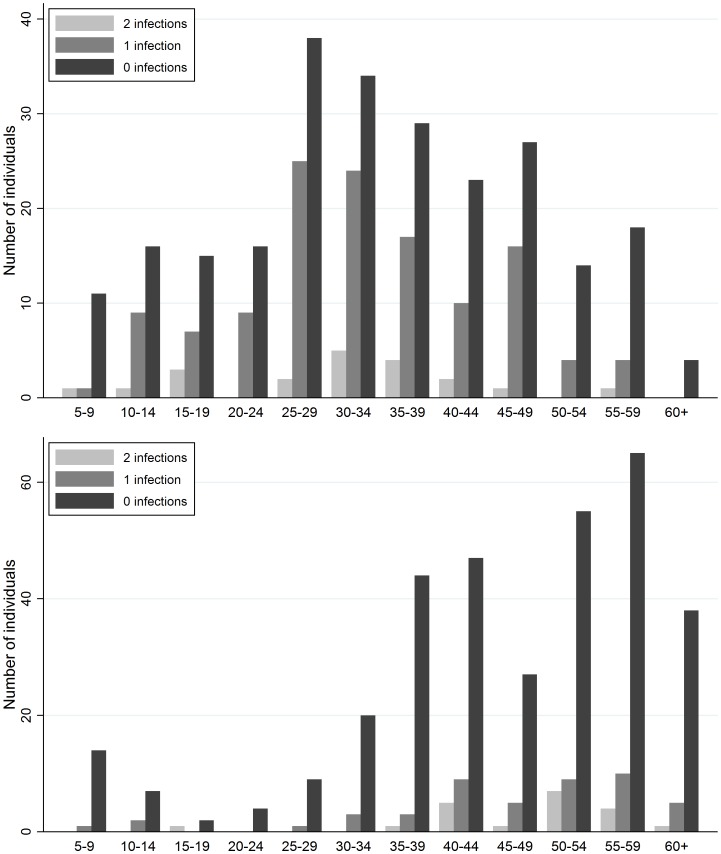
Distribution of incident *S. japonicum* infections by age in cohorts 1 (top) and 2 (bottom). Incident *S. japonicum* infections were measured at two follow-up points (2002 and 2006 in cohort 1, 2008 and 2010 in cohort 2). All participants were tested for *S. japonicum* at enrollment (2000 in cohort 1, 2007 in cohort 2) and all infections were promptly treated with praziquantel.

There were 21 and 20 individuals infected with *S. japonicum* at both *T_1_* and *T_2_* in cohorts 1 and 2, respectively (Table 3). Consecutive *S. japonicum* infections at follow-up were 3 and 7 times more common among those who were infected with *S. japonicum* at *T_0_* than those who were uninfected at *T_0_* in cohorts 1 and 2, respectively. Individuals that were infected at *T_1_* and *T_2_* were not demographically distinct from the cohorts as a whole. The age distributions of individuals with two consecutive infections to those with one or no infections at follow-up are similar (Figure 1). Among those with infections at *T_1_* and *T_2_*, mean age at enrollment was 30.1 (range 5–56) and 48.2 (18–63) in cohorts 1 and 2, respectively. In cohort 1, 11 of the 21 twice-infected individuals at follow-up were female and in cohort 2, 8 of 20 were female.

**Table 3:** The distribution of *S. japonicum* infections over time by baseline infection status.

	N	Infected at *T_1_* (%)	Infected at *T_2_* (%)	Infected at *T_1_* and *T_2_* (%)
**Cohort 1** *				
Infected at baseline				
No	222	56 (25.2)	11 (5.0)	5 (2.3)
Yes	202	83 (41.1)	30 (14.9)	16 (7.9)
**Cohort 2** †				
Infected at baseline				
No	315	21 (6.7)	20 (6.3)	7 (2.2)
Yes	85	23 (27.1)	24 (28.2)	13 (15.3)

* Cohort 1 is composed of people from 10 villages in Xichang County where schistosomiasis was endemic. Participants were tested for *S. japonicum* infection in 2000 (*T_0_*), 2002 (*T_1_*) and 2006 (*T_2_*).

† Cohort 2 is composed of people from 27 villages in two counties where schistosomiasis reemerged following reduction of *S. japonicum* infection prevalence below 1%. Participants were tested for *S. japonicum* infection in 2007 (*T_0_*), 2008 (*T_1_*) and 2010 (*T_2_*).

The observed fraction of the population with two consecutive *S. japonicum* infections was 1.48 times greater than expected in cohort 1, and 5.82 times greater than expected in cohort 2 (Table 4). This concentration of repeated *S. japonicum* infections in the same individuals is very unlikely due to chance (p = 0.00051 and p = 6.6×10^−12^ in cohorts 1 and 2, respectively). When we accounted for *S. japonicum* cercarial exposure, the ratios declined to 1.30 and 2.06 in cohorts 1 and 2, respectively. The excess of individuals with repeated *S. japonicum* infection, even when accounting for exposure, is highly unlikely due to chance in cohort 2 (p = 0.00056) and unlikely due to chance in cohort 1 (p = 0.013).

**Table 4:** The observed and predicted proportion of the population with two consecutive *S. japonicum* infections.

	Observed	Expected	Ratio (Obs./Exp.)	p-value‡
Cohort 1, simple prediction model*	5.07%	3.41%	1.48	0.00051
Cohort 1, exposure based prediction model†	5.07%	3.90%	1.30	0.013
Cohort 2, simple prediction model*	3.46%	0.59%	5.82	6.6×10^−12^
Cohort 2, exposure-based prediction model†	3.46%	1.68%	2.06	0.00056

* The expected prevalence of two consecutive infections was estimated based on the prevalence of infections at *T_1_* and *T_2_*.

† The expected prevalence of two consecutive infections was estimated accounting for *S. japonicum* exposure. The infection prediction model included water contact minutes by month and activity for all measures for which at least 20% of cohort participants reported exposure, age, sex, baseline village infection prevalence, county and year of infection test. Prediction models were fit separately for each cohort.

‡ P-values were estimated assuming the number of individuals with two consecutive infections follows a binomial distribution, 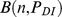 where 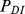 is equal to the expected prevalence of two consecutive infections and 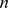 is equal to the number of individuals in the full population. Thus the p-value is that of a two-sided, one-sample test assuming the probability of double-infections is equal to *P_DI_*.

## Discussion

In two cohorts from two geographically distinct environments, *S. japonicum* infections repeatedly occurred in the same individuals over time, following treatment with praziquantel. This clustering of infections occurred even when accounting for exposure, and clustering was particularly strong in cohort 2, a population with low overall infection prevalence and intensity. These findings suggest there exists a subset of individuals within the general population that is particularly vulnerable to *S. japonicum* infection. Alternatively, this subset of individuals may have uncured infections due to non-compliance or treatment failure. This has important implications for disease surveillance: individuals with a history of *S. japonicum* infection may serve as appropriate targets for infection monitoring and treatment in low-prevalence environments. In addition, our findings provide evidence for host susceptibility to helminth infections – suggesting some individuals may be more vulnerable to infection given equivalent exposures.

It is possible that individuals who are repeatedly infected with helminthes are simply the most highly exposed individuals in the population. Cercarial exposure is a well-documented determinant of *S. japonicum* infection [37]–[40]. We found that the ratio of observed to expected prevalence of consecutive infections exceeded unity using an exposure-blind prediction model. This ratio was lower when we included exposure in the prediction models, but still exceeded unity. This suggests some individuals may be repeatedly infected due to their high cercarial exposure, but exposure does not fully explain this phenomenon. *S. japonicum* exposure is challenging to assess due to the difficulties in quantifying daily human behaviors and the absence of practical methods for directly measuring cercarial concentration, and our prediction models are limited by our ability to accurately measure cercarial exposure. However, the imperfections of our exposure measures are likely offset by the use of an aggressive, data adaptive algorithm to predict *S. japonicum* infection using over 25 exposure variables. Over-fitting is possible when using such methods, which, in this case, would have a conservative impact on our estimates, pushing observed to expected ratios closer to unity. Therefore, exposure alone is unlikely to explain the observed concentration of repeated schistosomiasis infections in a subset of the population.

More likely, individuals who are repeatedly infected with helminthes may be those who have a sufficiently elevated combination of susceptibility and exposure. We explored the clustering of infections within certain individuals from a mechanistic perspective by postulating that an individual's worm burden, *w*, accumulated from exposures subsequent to successful praziquantel treatment, can be described at the end of one or more infection seasons as a result of that individual's cumulative exposure to cercariae, *E*, and the subsequent penetration and development of a fraction of these cercarial hits, *α*, into adult parasites. That is, *w = αE* where *E* is composed of two elements, water contact, *S*, and cercarial concentration, *C.* The parameter *α*, reflecting host susceptibility, is assumed to be a stable property of each individual in the village population and the distribution of C is assumed to be a village property shared by all inhabitants. The water contact measurements described above and cercarial bioassay data collected in conjunction with the prior studies of cohort 1 [27], [37]suggest that the population distribution of *E* is strongly right skewed as is generally observed to be the case for distributions of *w*.

**Figure 2:**
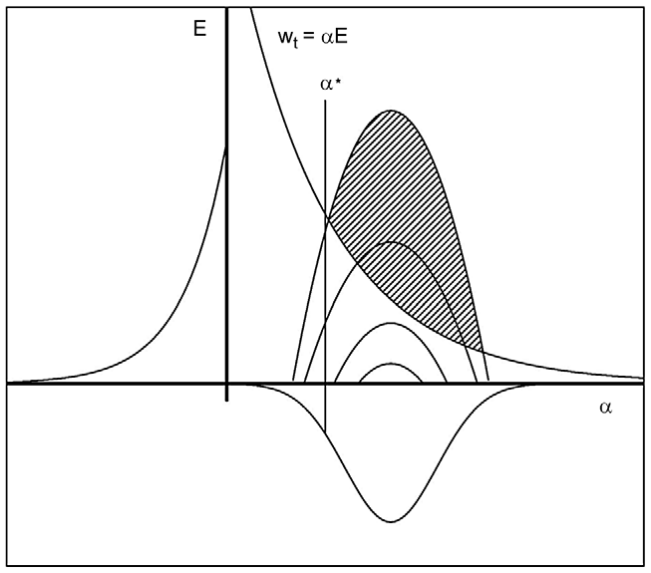
The marginal distributions of exposure and host susceptibility, together with contours of their joint distribution. The y-axis shows exposure, *E*, and the x-axis shows host susceptibility, *α*. The line *w_T_ = αE* describes the threshold of detectable infections: the minimum worm burden in an individual detectable by currently available assays. The shaded area depicts those combinations of *α* and *E* producing infection intensities above this lower limit of detection, w_t_. The fraction of the population susceptible to infection at or above this threshold is that lying to the right of the line *α* = α*.

Assuming that the distributions of exposure and susceptibility in a population are independent, their joint distribution is depicted in Figure 2. The marginal distribution of exposures, *f(E)*, is for illustrative purposes shown as a negative exponential distribution since multiple cercarial hits are thought to be necessary to lead to a single adult worm. Also for illustration, the marginal distribution of susceptibility, *h(α)*, is shown as symmetric. The line *w_T_ = αE* is the threshold of infections that are epidemiologically visible which we define here as the minimum worm burden necessary to produce eggs at the lower limit of detection by a combination of the miracidia hatching test and the Kato-Katz method. The fraction of the population susceptible to infection at or above this threshold is that lying to the right of the line *α* = α*. That is, the probability of an exposure leading to a diagnosis of infection for an individual with an *α* less than α* is essentially zero given the maximum cercarial exposure in this hypothetical environment. The shaded area depicts the set of exposure-susceptibility combinations that produce detectable infections.

Specification of the two marginal distributions allows the calculation of the distribution of their product, that is, the distribution of worm burden in the population. However, the point here is that, at least in this generic example, the proportion of the population at risk for infection is less than the entire population. That is, the number of individuals susceptible to infection, 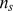, in this environment is:
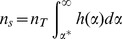
Where 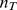 is the total population size. Hence, if 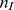 is the observed number of infections, the ratio of prevalence of infection in the susceptible population to the total population is:
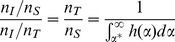
which is always equal to or greater than unity.

Returning to the re-infection issue, suppose the population is exposed in an unchanging environment, treated annually with praziquantel at *T* = 0, *T* = 1, and *T* = 2, and infection assessed at the end of year 1 and year 2. Since the same population is at risk of infection with the same marginal distribution of exposure in both years, and this population is less than the entire population, the observed number of repeated infections will be greater than that expected based on infections occurring randomly in the entire population. It follows that the ratio of observed re-infections to the expected number, if distributed randomly in the entire population, is simply the square of the foregoing equation:
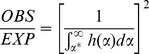
Moreover, as the fraction of exposure-susceptibility combinations that produce infection decreases, α* and this ratio both increase. Hence, the simple model of the infection process with individual differences in susceptibility to infection, depicted in Figure 2,provides a heuristic explanation of the epidemiological finding that the ratio of observed to expected re-infections increases as prevalence of infection decreases. Clearly, more refined analyses are possible that address a more rigorous definition of α*, take distributional assumptions into account, or explore the effect of variability in individual water contact. We will further address these and related determinants of transmission in the low-risk environment via an individually-based stochastic model which will be the subject of a future report. In addition, it is possible to estimate the proportion of susceptibles in a population via a statistical innovation using a model selection procedure like SuperLearner in the context of a latent mixture model, where the susceptibility status is latent – an approach that we will pursue in the future.

The factors that govern *α* are not fully characterized for schistosomiasis or other helminthiases. However, there is substantial evidence that immune function, particularly the ability to mount antigen-specific IgE response, can confer host resistance to schistosomiasis as well as other helminthiases [10], [11], [41]–[43]. Immune response is likely attributable to a combination of past exposure, treatment and host genetics [16], [44]–[46]. Physical characteristics such as skin thickness may also play a role in determining host resistance or susceptibility. As these genetic and immunological pathways are further elucidated, the definition of *α* may be further refined.

Alternatively, it is possible that the individuals who appear to be repeatedly infected with *S. japonicum* do not have new infections, but instead have residual, uncured infections that persist despite treatment. Praziquantel is the primary drug used to treat schistosomiasis infections, and resistance is an ongoing concern, particularly in areas where the drug has been used extensively. In China, praziquantel has been widely administered since the 1990s through mass and targeted treatment campaigns. Currently, there is no evidence of population-level resistance to *S. japonicum*, *S. haematobium* or *S. mansoni*, but praziquantel resistant laboratory isolates have been identified [47]–[51]. It is possible that praziquantel kills some but not all parasites, resulting in an incomplete cure. Repeated dosing with praziquantel may enhance treatment efficacy, particularly for individuals with high infection intensities [52]. While infection intensities in our two cohorts were generally low, we cannot rule out the possibility that what appear to be repeated infections are, in fact, infections that were not cured by praziquantel treatment.

Uncured *S. japonicum* infection may also be the result of poor adherence to drug treatment. As schistosomiasis morbidity declines, it is possible that so too do the perceived risks of infection and willingness to take praziquantel. Praziquantel has an excellent safety record and is appropriate for mass drug distribution, even in very young populations [53] but the drug has a bitter taste and can cause transient side effects, including nausea and dizziness. In a recent survey, 33% of people said such side effects impacted their ability to work [49]. We have found a high degree of self-reported treatment adherence (>90%) in surveys of 236 people drawn from the same villages as cohort 1 (surveyed in 2007) and 686 people drawn from the same villages as cohort 2 (surveyed in 2008), but other studies have documented poor compliance with mass-treatment campaigns for helminthiasis [54], [55]. Our findings underscore the importance of continued monitoring of treatment effectiveness, including both drug resistance and population perceptions of the risks and benefits of treatment. Methods capable of distinguishing new from residual infections could advance our understanding of treatment efficacy and drug adherence.

Our findings underscore surveillance challenges in areas where worm burdens are low. While individuals with high worm burdens have the potential to contribute a large number of future infections, our prior work suggests that even modest parasite inputs are sufficient to sustain schistosomiasis transmission [7]. In China, surveillance and elimination efforts are made more complex as there are at least 40 competent mammalian host species for *S. japonicum*, and bovines are suspected to be key reservoirs in some areas [56]. Thus the ability to identify humans and, in the case of *S. japonicum*, other mammalian hosts with low-intensity helminth infections may be crucial to efforts to prevent the reemergence of helminth infections in areas where disease control efforts have successfully lowered infections and morbidity. Many of the individuals who tested positive for *S. japonicum* in our study had worm burdens below the limit of detection of the Kato-Katz assay, the schistosomiasis diagnostic method recommended by the World Health Organization [6]. Immunoassays generally have high sensitivity, but it can be difficult to distinguish past from current infections, which is particularly problematic when attempting to identify residual infections in regions with previously high infection prevalence and intensity [57], [58]. While new methods offer promise [59], the current lack of practical, highly sensitive diagnostics is a barrier to the long-term control of helminthiases [1], [60].

As China aims to eliminate schistosomiasis and global efforts are launched to eliminate a number of helminthiases, the success of such efforts may hinge, in part, on the ability to identify reservoirs of infection and reduce the potential of such reservoirs to generate future infections. Our findings suggest that there exist an identifiable, high-risk subpopulation for *S. japonicum* infection. Due to high exposure, host susceptibility or treatment failure, these individuals are potential future reservoirs of *S. japonicum*. Further, as infection prevalence declines, and with it, cercarial exposure, we expect the fraction of the population that is susceptible to *S. japonicum* infection to decline. Thus, as regions approach disease control goals, targeted interventions may prove efficient and effective. In low-prevalence regions, individuals who test positive for *S. japonicum* should be tested regularly and provided pharmaceutical treatment and transmission-blocking interventions such as improved household latrines [56], [61].

## Supporting Information

Text S1 **Supporting Information S1.** Example of a questionnaire used to assess water contact behaviors. **Supporting Information S2.** STROBE Checklist. **Figure S1.** Selection and retention of cohort 1. **Figure S2.** Selection and retention of cohort 2. **Table S1.** Comparison of participants from cohorts 1 and 2 with complete data to those who were lost to follow-up. **Table S2.** Comparison of participants from cohort 2 with complete vs. partial water contact questionnaire survey data.
(PDF)
