# Supplementary material for: Repeated Schistosoma japonicum Infection Following Treatment in Two Cohorts: Evidence for Host Susceptibility to Helminthiasis?
Source: PLoS Negl Trop Dis. 2013 Mar 7;7(3):e2098. doi: 10.1371/journal.pntd.0002098 (PMC3591324; doi:10.1371/journal.pntd.0002098)
Supplement: Text S1 — Supporting Information S1. Example of a questionnaire used to assess water contact behaviors. Supporting Information S2. STROBE Checklist. Figure S1. Selection and retention of cohort 1. Figure S2. Selection and retention of cohort 2. Table S1. Comparison of participants from cohorts 1 and 2 with complete data to those who were lost to follow-up. Table S2. Comparison of participants from cohort 2 with complete vs. partial water contact questionnaire survey data. (PDF) [file pntd.0002098.s001.pdf]

Supporting Information S1. Example of a questionnaire used to assess water contact behaviors.

**Activity**	In the past half month, did your skin contact irrigation, pond or stream water while [activity]?<br>1. Yes<br>0. No	If yes:<br>Where did you do [activity]?<br>For each location, record the grid location (i.e. A4).		How many times in the last half month did you do [activity] in this location?	How many minutes did you usually do [activity] each time?
Washing clothes		1			
		2			
		3			
Washing agricultural tools		1			
		2			
		3			
Washing hands or feet		1			
		2			
		3			
Playing or swimming		1			
		2			
		3			
Ditch cleaning or water diverting		1			
		2			
		3			
Planting rice		1			
		2			
		3			
Harvesting rice		1			
		2			
		3			
Fishing		1			
		2			
		3			
Washing vegetables		1			
		2			
		3			

This questionnaire was used for cohort 2. Each participant was asked to answer these questions using a gridded map of his/her village (below). The map is intended to help participants recall water contact, and to allow for evaluation of spatial patterns of water contact, described in detail elsewhere [1]. Participants indicate where on the map each activity occurred and the interviewer records the coordinates (e.g. E16).

Supporting Information S1 (continued). Example high-resolution gridded map of a study village to be used with the water contact questionnaire. Irrigation ditches (green dots) and houses (white squares) are shown.

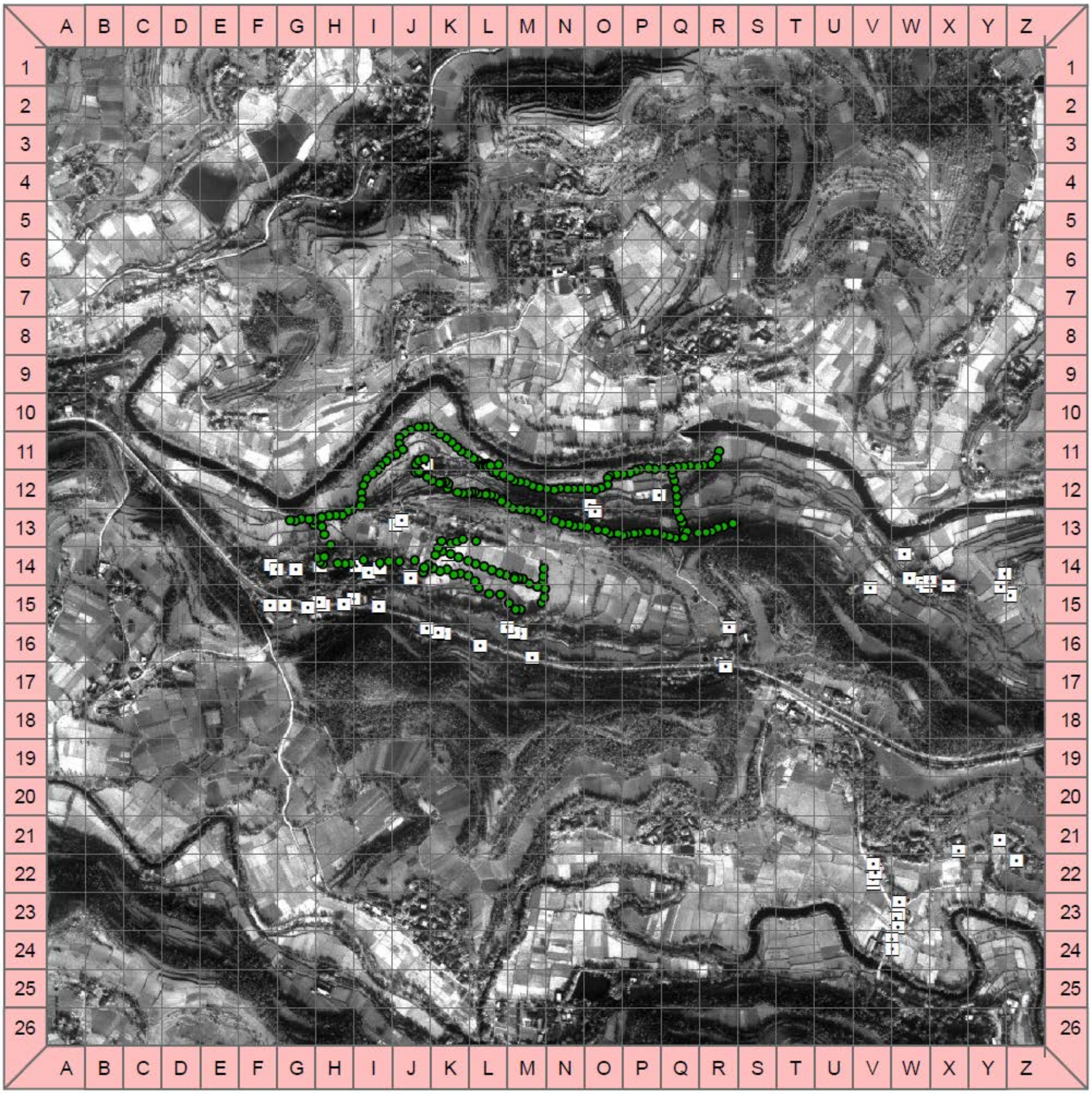

References

1. Seto EY, Lee YJ, Liang S, Zhong B (2007) Individual and village-level study of water contact patterns and *Schistosoma japonicum* infection in mountainous rural China. Trop Med Int Health 12: 1199-1209.

Figure S1. Selection and retention of cohort 1.

Baseline infection (T_0): *S. japonicum* infection surveys conducted in 10 villages in Xichang County, Sichuan, China in November 2000.* All residents, age 4-60, targeted. 1,801 people tested.

Exposure assessment: 25% random sample of participants, stratified by occupation and village, interviewed about water contact behaviors, November 2000. 566 people interviewed.

Initial cohort: 551 people in 10 villages who completed the infection and water contact surveys

↓

Follow-up infection (T_1): *S. japonicum* infection surveys conducted, November 2002. 490 people in 10 villages.

↓

Follow-up infection (T_2): *S. japonicum* infection surveys conducted, November 2006. 424 people in 10 villages

*In 2000, *S. japonicum* infection and exposure surveys were conducted in 20 villages in Xichang county [1]. Ten villages with high infection prevalence (range: 12.9, 72.3%) were selected for longitudinal follow-up. This figure describes participants from the 10 villages that were followed longitudinally.

References

1. Spear RC, Seto E, Liang S, Birkner M, Hubbard A, Qiu D, Yang C, Zhong B, Xu F, Gu X, Davis GM (2004) Factors influencing the transmission of *Schistosoma japonicum* in the mountains of Sichuan province of China. Am J Trop Med Hyg 70: 48-56.

Figure S2. Selection and retention of cohort 2.

Baseline infection (T_0): *S. japonicum* infection surveys conducted in 36 villages in 2 counties where schistosomiasis reemerged following reduction of human infection prevalence below 1%.* All residents, age 6 to 65 targeted in November and December 2007. 2,110 people tested.

Exposure assessment: All people who tested positive for *S. japonicum* infection (169 people) and, for each infected person, five randomly drawn people from the same village that tested negative for *S. japonicum* infection (626 people) were selected for monthly interviews to assess water contact behaviors.† Interviews were conducted June-October 2008. 781 people interviewed.

Initial cohort: 781 people in 28 villages in two counties who participated in the infection and water contact surveys.

↓

Follow-up infection (T_1): *S. japonicum* infection surveys conducted, November and December 2008. 599 people in 27 villages.

↓

Follow-up infection (T_2): *S. japonicum* infection surveys conducted, November and December 2010. 400 people in 27 villages.

*In 2007, *S. japonicum* infection surveys were conducted in 53 villages in three counties where schistosomiasis had reemerged [1]. The magnitude 7.9 earthquake that occurred in Sichuan May 12, 2008, severely impacted one of the selected counties. Due to extensive recovery efforts in this county, we limited follow-up to the two other counties. This figure describes participants from the two counties that were followed longitudinally.
†In villages where greater than 1 in 6 people were infected, 5:1 matching could not be attained. All individuals who tested negative for *S. japonicum* infection in such villages were selected. Exposure assessment was not conducted in the 8 villages where no *S. japonicum* infections were detected.

References

1. Carlton EJ, Bates MN, Zhong B, Seto EY, Spear RC (2011) Evaluation of mammalian and intermediate host surveillance methods for detecting schistosomiasis reemergence in southwest China. PLoS Negl Trop Dis 5: e987.

Table S1. Comparison of participants from cohorts 1 and 2 with complete data to those who were enrolled but subsequently lost to follow-up.

	Cohort 1		Cohort 2	
	Complete	Incomplete	Complete	Incomplete
Number of individuals	424	127	400	381
Percent positive for *S. japonicum* at enrollment	47.6	42.5	21.3	21.3
Mean infection intensity at enrollment (SE)	53.6 (10.8)	45.5 (11.8)	5.2 (2.2)	5.3 (1.3)
Mean age at enrollment (SE)	31.1 (0.7)	25.1 (1.4)	45.6 (0.7)	38.7 (0.9)
Percent Female	51.9	47.2	57.3	45.7
% reporting any water contact	95.0	87.4	95.0	87.1
Mean total water contact hours (SE)	59.6 (2.7)	48.4 (5.9)	42.6 (2.4)	29.6 (1.9)

EPG: Eggs per gram of stool; SE: Standard error
All participants described in the table were tested for *S. japonicum* infection at enrollment and completed water contact questionnaires. Individuals with complete follow-up were additionally tested for *S. japonicum* infection at two follow-up periods.

Table S2. Comparison of participants from cohort 2 with complete vs. partial water contact questionnaire survey data.

	Complete (n=316)	Incomplete (n=84)*	P-value†
Mean age (SE)	47.9 (0.7)	41.6 (2.0)	0.0002
Percent female	56.6	59.5	0.636
Percent from county 1	65.8	51.2	0.014
Percent positive for *S. japonicum* in 2007	21.8	19.0	0.579
Mean EPG in 2007 (SE)	5.7 (2.8)	3.1 (1.2)	0.598

EPG: Eggs per gram of stool; SE: Standard error

*Participants with incomplete water contact data include 71 participants who did not complete every monthly interview (but completed at least 1 interview), and 13 participants who participated in all six water contact interviews but had incomplete data from one or more months.

†P-values were calculated using Pearson Chi^2 for binary variables, the student's t-test for age (which is normally distributed), and the Kruskal-Wallis rank sum test for EPG (which is right-skewed).
